# Early urodynamic profiles predict lower urinary tract recovery after spinal cord injury in female rats

**DOI:** 10.14814/phy2.71101

**Published:** 2026-09-09

**Authors:** Behnaz Afrashteh, Steafan Khan, Rabeya Zinnat Adury, Damiano Angoli, Zachary C. Danziger

**Affiliations:** ^1^ Division of Physical Therapy, Department of Rehabilitation Medicine Emory University Atlanta Georgia USA; ^2^ Department of Biomedical Engineering Florida International University Miami Florida USA; ^3^ Department of Applied Physiology and Kinesiology University of Florida Gainesville Florida USA; ^4^ Wallace H. Coulter Department of Biomedical Engineering Emory University and Georgia Institute of Technology Atlanta Georgia USA

**Keywords:** neurogenic bladder, predictive modeling, recovery phenotypes, spinal cord injury, urodynamics

## Abstract

Bladder dysfunction is a major complication after spinal cord injury (SCI) and effective treatment remains challenging. Neurogenic bladder can be difficult to treat because of its heterogeneous and dynamic symptoms. We aimed to (1) characterize heterogeneity in bladder recovery from acute to chronic phases and (2) predict chronic urinary function using early urodynamic parameters. We performed continuous longitudinal urodynamic monitoring in 10 complete T9 SCI and 10 control rats over 4 weeks. A novel metabolic cage system enabled 24/7 tracking of voiding behavior. Key parameters, including voiding efficiency (VE), post‐void residual (PVR), and ureter flow rate (UFR), were measured continuously throughout recovery. Unsupervised clustering identified three distinct spinally mediated reflexive voiding phenotypes in the chronic phase (days 20–30), defined by VE and PVR: good (robust efficient voiding), moderate (partial voiding efficiency), and poor (minimal voiding efficiency). No single acute‐phase urodynamic parameter (3–10 days post‐injury) differed significantly between phenotypes. In contrast, multivariate generalized linear models incorporating parameter interactions, particularly VV × VE and UFR × VE^2^, predicted chronic phenotype with high accuracy (*R* ^2^ = 0.90, *p* < 0.001). These findings underscore the prognostic importance of interactions among early urodynamic features in determining the severity of long‐term neurogenic bladder outcomes after SCI and identify a critical window for individualized intervention.

## Graphical abstract

After spinal injury multiple urological phenotypes emerge. Multivariate analysis of Continuously collected urodynamic data from early after the injury can predict the chronic phase phenotype.

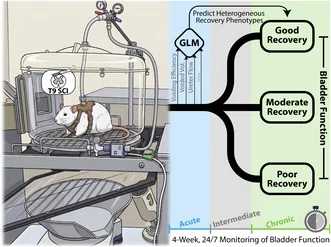

## 1 INTRODUCTION

Neurogenic bladder, affecting approximately 80% of individuals with SCI, profoundly influences long‐term quality of life and survival (Manack et al., 2011; Panicker et al., 2015; Taweel & Seyam, 2015). A major challenge in developing effective treatments is the difficulty in early diagnosis due to marked heterogeneity in lower urinary tract (LUT) recovery profiles (Saito et al., 2021; Wyndaele, 2016). Recovery progresses slowly over an extended timeline: an initial spinal shock phase in which detrusor areflexia persists for 6–12 weeks post‐injury (Ditunno et al., 2004), followed by a reorganization phase in which spinal pathways connecting bladder afferents to motor neurons are progressively reactivated and voiding re‐emerges as a spinally mediated reflexive pattern analogous to neonatal micturition, in which the bladder empties reflexively upon reaching a threshold intravesical pressure (de Groat & Yoshimura, 2006; Groen et al., 2016). Critically, this reorganization is not efficient and does not represent restoration of pre‐injury LUT function; rather, it reflects a reversion to a primitive spinal reflex circuit. As the chronic phase develops (beyond 12 months post‐injury), differential spinal neuroplasticity, progressive C‐fiber afferent sensitization, and structural bladder remodeling interact to amplify individual differences in spinally mediated reflexive patterns, giving rise to heterogeneous long‐term LUT trajectories: from progressive detrusor hyperreflexia, detrusor‐sphincter dyssynergia, and bladder wall hypertrophy at one extreme, to spontaneous efficient reflexive voiding at the other (Ahmed et al., 2006; Anderson, 2004; de Groat, 1997).

Current diagnostic approaches, however, fail to capture these individual differences. Acute‐phase management prioritizes medical stabilization and catheterization, delaying comprehensive urodynamic assessment until weeks or months post‐injury (Ginsberg et al., 2021; Stöhrer et al., 2009), missing the critical early window when interventions could prevent irreversible secondary bladder damage. Moreover, interpreting urodynamic data is another diagnostic challenge because the bladder functions as a synchronized neuromuscular system where outcomes depend on coordinated multivariate interactions rather than individual parameter values.

Machine learning (ML) can detect these complex patterns and reveal early indicators of chronic dysfunction hidden within acute‐phase profiles (Dietz et al., 2022; Kato et al., 2024; Maki et al., 2024). However, its application to neurogenic bladder is limited. Studies like Pavese et al. (2016) and Kitamura et al. (2025) use early clinical scores to predict bladder function at discharge or one year after injury, without urodynamic recording of the intervening period. Rat SCI models replicate key features of human neurogenic bladder and enable continuous monitoring under control conditions, yet no preclinical study has tracked LUT function across the entire recovery timeline while examining interindividual variability (Kjell & Olson, 2016; Shen et al., 2021).

To address this knowledge gap, we conducted continuous longitudinal urodynamic monitoring in rats following complete T9 thoracic transection. Using a custom metabolic cage system that enabled 24/7 automated recording of every voiding event across active and rest phases of the diurnal cycle (Angoli et al., 2020), we aimed to characterize LUT reorganization after SCI and to test whether early multivariate urodynamic patterns, using a generalized linear model (GLM) framework (Burnham & Anderson, 2003), can predict long‐term bladder outcomes. We found that early multivariate urodynamic interactions accurately predict chronic voiding phenotype, providing a basis for personalized intervention during the period when the spinal cord and bladder are most adaptable.

## 2 MATERIALS AND METHODS

### 2.1 Animals

Adult female Sprague–Dawley rats (*n* = 20; 3–5 months; 334.6 ± 10.6 g) were housed individually in metabolic cages under controlled temperature, humidity, and a 12‐h light/dark cycle, with food (Rodent Diet 5053, LabDiet, St. Louis, MO, USA) and water ad libitum. All procedures were approved by the Florida International University Institutional Animal Care and Use Committee.

### 2.2 Surgical procedures

Rats (*n* = 20; 10 intact and 10 SCI) were initially anesthetized using inhalation of isoflurane (1.5% in oxygen), followed by ketamine (90 mg/kg) and xylazine (10 mg/kg) injection. After exposing the bladder, a 3‐Fr silicone catheter with a custom‐designed 4‐mm fenestrated tip was implanted in all animals and fastened to the bladder dome. Following catheter implantation, complete T9 spinal cord transection was performed in the 10 rats assigned to the SCI group: a laminectomy was performed at T8/T9 while preserving the facet joints, and the dura and spinal cord were completely transected at the T9 level, with a sterile Gelfoam sponge placed between the cut ends. The 10 intact control rats underwent identical bladder catheter implantation but did not receive a laminectomy or spinal cord transection; no sham spinal surgery was performed. Both groups were therefore matched for LUT surgical exposure, and the intact group served as a normal physiological reference for urodynamic parameters. Post‐operative care included subcutaneous injections of buprenorphine (0.03 mg/kg) and cefazolin (33.3 mg/kg) administered twice daily for 5 consecutive days. To mitigate the ongoing risk of urinary tract infections (UTIs) and systemic sepsis associated with neurogenic bladder retention, the drinking water was supplemented with enrofloxacin (0.5 mg/mL) throughout the entire experimental period.

### 2.3 SCI lesion verification and inclusion criteria

Completeness of spinal cord transection was verified behaviorally during the acute postinjury period. All SCI rats demonstrated complete bilateral hindlimb paralysis with absent voluntary motor function below the T9 injury level throughout the first 5 days post‐SCI, consistent with complete supraspinal disconnection. Rats exhibiting any voluntary hindlimb movement, weight‐bearing, or coordinated stepping during this period would have been excluded as incomplete injuries. However, all rats in this study met criteria for complete transection and were retained for analysis.

### 2.4 Urodynamic assessment

Continuous urodynamic recordings were obtained in intact rats (*n* = 10) beginning 3 days after catheter implantation, and in SCI rats (*n* = 10) once spontaneous voiding had been regained (typically 3–7 days after injury).

The urodynamic assessment system was set up based on our previously published study describing a method for estimating postvoid residual volume without direct measurement during serial cystometrograms (Angoli et al., 2020). In brief, we utilized modified Technoplast metabolic cages with standard metal mesh floors. Below the floor were angled, laser‐etched fine wire mesh sheets (Med Associates Inc.) that allowed urine to pass through instantly while solid feces were blocked and rolled down the grade to avoid absorbing or deflecting falling urine. Digital scales (HCB 302, Adam Equipment) placed below the cage floor continuously measured urine weight at 1 Hz. Bladder pressure was monitored via the implanted catheter connected to an inline pressure transducer (TD23, Transonic) and Trans Bridge amplifier (TBM4M, World Precision Instruments), at 40 Hz through a data acquisition system (USB‐6218, National Instruments).

Without saline infusion, residual urine was emptied every 12 h via a stopcock interfaced with the fluid commutator, while bladder pressure and voiding were simultaneously recorded. As needed, but no more frequently than every other day, < 1 mL of Taurolidine‐Citrate Solution (TCS; taurolidine 1.35%/sodium citrate 4%; Braintree Scientific, Braintree, MA) was gently instilled into the catheter assembly to maintain patency and prevent intraluminal blood clotting, crystalline occlusion, and bacterial biofilm formation (Liu et al., 2014).

### 2.5 Urodynamic parameters

From the continuous pressure and voided‐volume traces, the following parameters were extracted. A contraction was classified as a voiding contraction if an increase in voided volume was registered on the collection scale. Maximum voiding pressure was defined as the peak intravesical pressure reached during a voiding contraction. The inter‐void interval was defined as the period between two consecutive voiding contractions. Within each inter‐void interval, non‐voiding contractions (NVCs) were identified as transient pressure rises exceeding 5 mmHg above baseline with no void occurring within 5 s before or 15 s after. NVC frequency was calculated by counting the NVCs in each inter‐void interval and averaging those counts for each 24‐h period; the first and last intervals of each 24‐h period, which were truncated by the period boundaries, were included in this average.

Using the residual volume measurements in 12‐h periods, along with the known water intake and voided volumes recorded during continuous urodynamic assessment, we applied our previously established estimation algorithm to calculate post‐void residual (PVR) and ureter flow rate (UFR) for each voiding event (Danziger & Grill, 2016). This approach was less invasive and minimized potential disruptions to normal bladder function throughout the experiment.

### 2.6 Data analysis

Data were analyzed in MATLAB v2023a (MathWorks) and Python (SciPy, pandas, matplotlib). Through a comprehensive processing pipeline. Raw bladder pressure signals underwent initial conditioning through a 4th order Butterworth filter (0.1–10 Hz bandpass) to eliminate artifacts and noise, with baseline drift correction using a 30‐s moving window average updated hourly. Data were collected from intact and SCI groups, with each animal contributing two 12‐h cycles (light and dark) per day. For SCI rats, data from the light and dark cycles were pooled for analysis, as no statistically significant diurnal variations were observed. In contrast, only data from the light cycle were used for the intact group because during that cycle we do not observe deliberately partial voids putatively used for social queuing (Angoli et al., 2020).

Data were analyzed within three predefined post‐injury phases: early (days 3–10), intermediate (days 10–20), and chronic (days 20–30) (Herrera et al., 2010; Mimata et al., 1993). Prior to analysis, outliers were removed at the level of individual events using the interquartile range method (values <Q1–1.5 × IQR or >Q3 + 1.5 × IQR). For each parameter, values were averaged across voiding cycles and across recording days within each phase, providing one mean value per animal per phase for statistical analysis. All available recording days within each phase window were included per animal, except for one intact animal that contributed only a single recording day to the chronic phase; this animal was excluded from chronic‐phase analyses (chronic phase, intact group: *n* = 9). Between‐group differences at each phase were assessed using Welch's ANOVA followed by the Games‐Howell post hoc test. Voiding parameters were compared among the Intact, Good, and Moderate phenotypes. The Poor phenotype (*n* = 1) was excluded from all statistical analyses. Data are presented as mean ± SD.

### 2.7 Clustering of SCI chronic phase recoveries

Distinct clusters of animals were identified with regards to rat's median VE and median PVR in the chronic phase of SCI recovery. The medians for each rat were computed from all their voids during the chronic phase. The medians were clustered using DBSCAN (Density‐Based Spatial Clustering of Applications with Noise) with a neighborhood of ϵ=0.3 and a minimum of one neighbor per core point. Confidence ellipsoids for the medians were computed via a bootstrapping procedure wherein each animal's chronic phase voids were randomly sampled with replacement 1000 times using the *bootstrp* function (MATLAB R2023b, Statistics and Machine Learning Toolbox; MathWorks, Natick, MA). The confidence ellipsoids were drawn to capture 90% of the variance of the bootstrapped medians.

#### 2.7.1 Aa. Exploratory data analysis: Predicting chronic VE from acute urodynamic parameters

A GLM was constructed to investigate whether the median chronic void efficiency (${\tilde{\mathrm{VE}}}_{c}$) of each rat could be accurately predicted from a subset of their median acute urodynamic parameters (Voided Volume (${\tilde{\mathrm{VV}}}_{a}$), Post Void Residual (${\tilde{\mathrm{PVR}}}_{a}$), Bladder Capacity (${\tilde{\mathrm{BC}}}_{a}$), Void Efficiency (${\tilde{\mathrm{VE}}}_{a}$), Inter‐Void Interval (${\tilde{\mathrm{IVI}}}_{a}$), and Ureter flow rate (${\tilde{\mathrm{UFR}}}_{a}$)). The GLM utilized a logit‐link function since the median chronic void efficiency (${\tilde{\mathrm{VE}}}_{c}$) was approximately logit‐normally distributed, and the GLM was constrained to have at most 3 terms to avoid overfitting. The general form of the GLM is depicted in (1).

(1) $${\tilde{\mathrm{VE}}}_{c}\approx \frac{e^{p_{0}+p_{1}{\tilde{x}}_{1}+p_{2}{\tilde{x}}_{2}}}{1+e^{p_{0}+p_{1}{\tilde{x}}_{1}+p_{2}{\tilde{x}}_{2}}}$$

where ${\tilde{\mathrm{VE}}}_{c}$ represents the estimate of median chronic Void Efficiency, $\left\{p_{0},p_{1},p_{2}\right\}$ are the model coefficients to be fit, and $\left\{\;{\tilde{x}}_{1},{\tilde{x}}_{2}\right\}$ is a two‐parameter subset from all the available median acute urodynamic parameters. To enhance the GLM's ability to capture nonlinear relationships in the data, the potential median acute urodynamic parameters were expanded to include squared terms and interactions (e.g., ${\tilde{{\mathrm{VV}}_{a}}}^{2},\tilde{V_{a}}\tilde{{\mathrm{PVR}}_{a}},\tilde{{\mathrm{VV}}_{a}}\tilde{{\mathrm{BC}}_{a}}\ldots$ etc.). This resulted in 78 $\left(12+\left(\frac{12}{2}\right)\right)$ possible median acute urodynamic parameters $\tilde{x}$ to use for prediction. The optimal $\left\{\;{\tilde{x}}_{1},{\tilde{x}}_{2}\right\}$ were identified as $\left\{\;\tilde{{\mathrm{VV}}_{a}}\cdot {\tilde{\mathrm{VE}}}_{a},{\tilde{\mathrm{UFR}}}_{a}\cdot {{\tilde{\mathrm{VE}}}^{2}}_{a}\right\}$ via a best subset selection procedure. All 3003 possible GLMs (pairwise combinations of the 78 potential parameters) were trained using the expanded acute urodynamic parameters from all 10 rats to predict the chronic void efficiency (${\tilde{\mathrm{VE}}}_{c}$). The coefficients $\left\{p_{0},p_{1},p_{2}\right\}$ for each candidate for GLM were calculated via iteratively reweighed least squares in MATLAB. The parameters $\left\{\;\tilde{{\mathrm{VV}}_{a}}\cdot {\tilde{\mathrm{VE}}}_{a},{\tilde{\mathrm{UFR}}}_{a}\cdot {{\tilde{\mathrm{VE}}}^{2}}_{a}\right\}$ resulted in the best fit GLM and were used for all final analyses (Shen et al., 2021). To estimate the GLM's predictive accuracy on unseen data, i.e., model validation, a leave‐one‐out cross validation was performed, wherein the GLM coefficients $\left\{p_{0},p_{1},p_{2}\right\}$ were computed utilizing data from only 9 of the 10 rats and the performance predicting the median chronic void efficiency (${\tilde{\mathrm{VE}}}_{c}$) of the left‐out rat was evaluated. This process was repeated 10 times, such that each rat was left out once. Due to the limited size of the dataset (*n* = 10), there was no testing set.

#### 2.7.2 Ab. Exploratory data analysis: Latent trajectories of SCI rat recoveries

To investigate whether the trajectories of recovery differed across the recovery phenotypes, each rat's median urodynamic parameters were computed in each phase (acute, intermediate, chronic) and projected into a low‐dimensional representation using Principal Components Analysis (e.g. Churchland et al., 2012). The median urodynamic parameters ($\tilde{\mathrm{VV}},\tilde{\mathrm{PVR}},\tilde{\mathrm{VE}},\tilde{\mathrm{IVI}},\tilde{\mathrm{UFR}}$) for each phase were expanded to include first order interactions and squared terms (BC was not included since it is linearly dependent). This resulted in a data matrix $D\in R^{30\times 20}$ (10 rats × 3 phases by 20 expanded urodynamic parameters) in which each dimension of the data was a urodynamic parameter, and the rows were rats across time. The data was normalized across rats and phases by centering each urodynamic parameter about its median and dividing each urodynamic parameter by its IQR. The Principal Components of the normalized dataset were computed. The first two PCs explained 76% of the variance in the data. A varimax rotation was applied to PCs 1 and 2 to simplify their structure. The median urodynamic parameters were projected onto the rotated principal components (RCs) and plotted to visualize the change in all urodynamic parameters throughout the time course of the rats' recoveries. Continuous trajectories were drawn by fitting a piece‐wise cubic interpolating polynomial to the median RC scores for each phase using the *interp1* function (MATLAB 2023b). Confidence ellipsoids for the median RC scores were estimated via a bootstrapping procedure wherein the RC scores for each animal's voids in each phase were randomly sampled with replacement 1000 times using the *bootstrp* function (MATLAB 2023b, Statistics and Machine Learning Toolbox). The confidence ellipsoids were drawn to capture 90% of the variance of the bootstrapped median RC scores.

## 3 RESULTS

### 3.1 Longitudinal analysis of voiding parameters across SCI and intact groups

**FIGURE 1:**
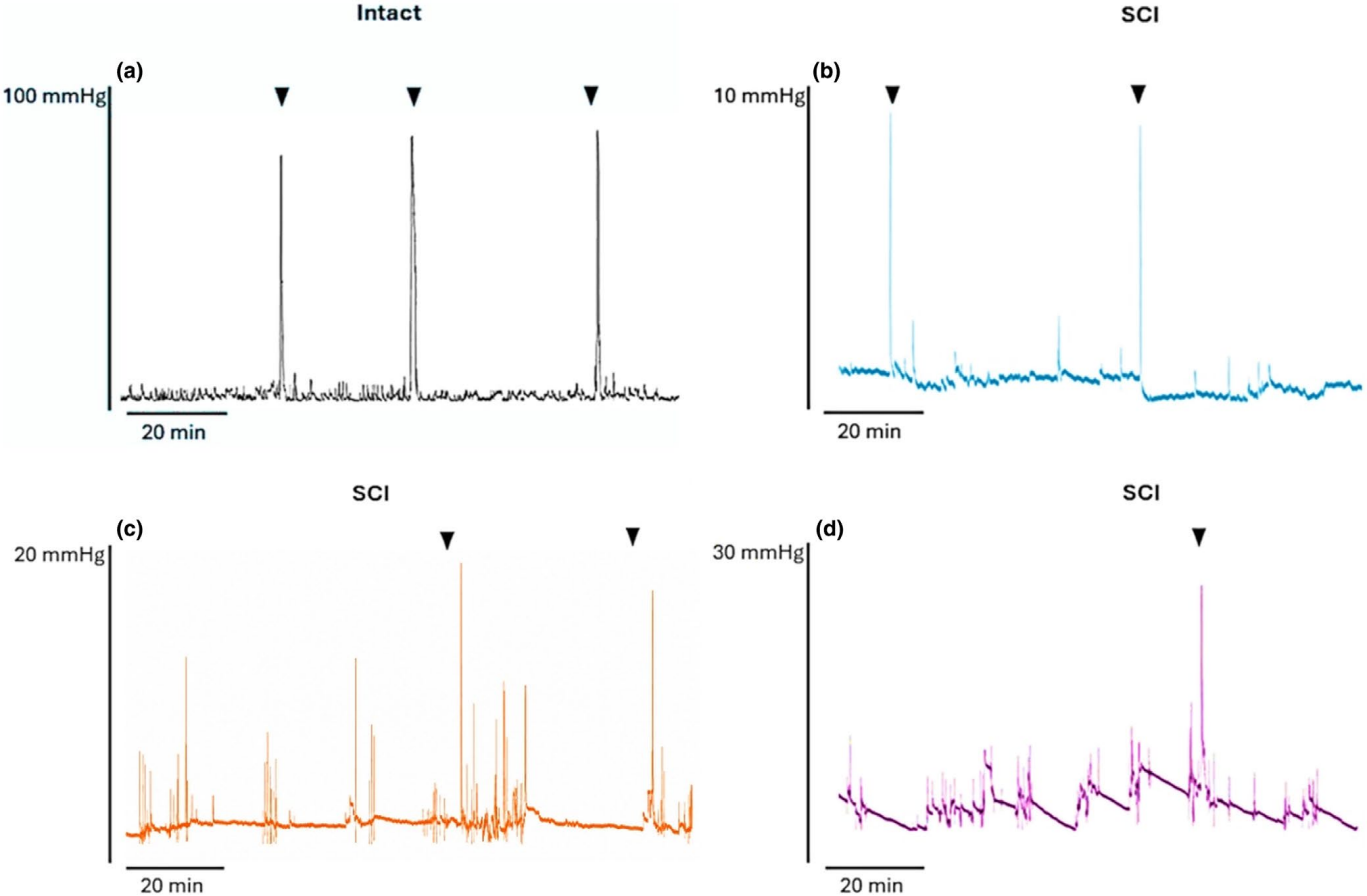
Representative continuous intravesical pressure recordings during the chronic phase. Intravesical pressure (mmHg) is plotted against time; void events are indicated by black triangles. Note that vertical (pressure) scale bars differ between panels (100, 10, 20, and 30 mmHg for a–d, respectively); horizontal scale bars, 20 min. (a) Intact rat, showing regular pressure rises corresponding to voiding events. (b–d) SCI rats, illustrating the heterogeneity of chronic voiding patterns, including elevated baseline pressure fluctuations, irregular voiding intervals, and variable peak pressures. Panels show representative examples of the Good (b), Moderate (c), and Poor (d) recovery phenotypes identified by clustering analysis (Figure 3).

Urodynamic data were collected from both intact and SCI rats housed in custom‐designed metabolic cages that allowed uninterrupted, continuous monitoring of bladder pressure (via an indwelling catheter) and voiding events (via a digital scale) for approximately 26 ± 4 days post‐injury in the SCI group and a comparable 25‐day period in the intact group. Representative bladder pressure traces are shown in Figure 1, illustrating the diverse voiding patterns observed among SCI rats in the chronic phase. These ranged from regular, rhythmic voiding contractions (Figure 1b) to dysregulated detrusor activity characterized by frequent non‐voiding contractions (NVCs) and unstable intravesical pressure during the intervoid intervals (Figure 1c,d).

**FIGURE 2:**
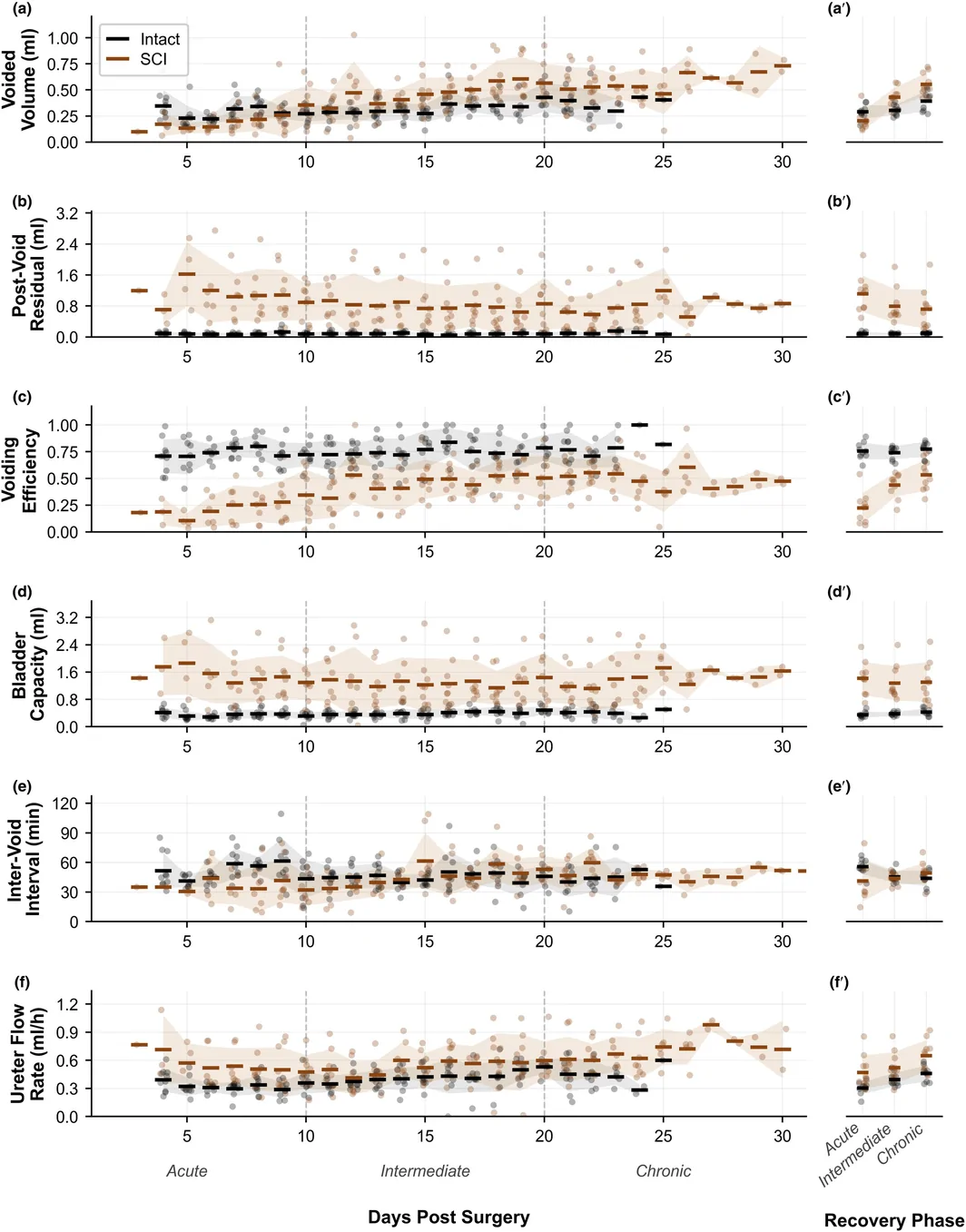
Urodynamic parameters across recovery phases in intact and SCI rats. (a–f) Time course of urodynamic parameters up to 30 days post‐surgery in intact (black; light cycle only) and SCI (brown) groups. (a′–f′) Phase‐wise summary statistics for each parameter. Data are expressed as mean ± SD.

To assess the progression of neurogenic bladder, urodynamic parameters were plotted longitudinally in the SCI group (from day 3 to 26 ± 4 days post‐injury) and compared to the intact group at corresponding time points (Figure 2a–f). VV and the inter‐void interval (IVI) were measured directly, and UFR and PVR were estimated to use a previously established optimization algorithm as detailed in the Methods section (Urodynamic Assessment) (Angoli et al., 2020; Danziger & Grill, 2016). Bladder capacity (BC) was calculated as the sum of estimated PVR and measured VV, and VE was determined as the ratio of VV to estimated BC. These parameters served as key indicators for monitoring bladder function over time. Notably, two critical markers of LUT function following SCI are high VE, reflecting effective reflex activation and coordinated detrusor‐sphincter function, and low PVR, indicating reduced bladder overdistension.

Longitudinal analysis of VE and PVR in the SCI group revealed a gradual improvement in VE and a reduction in PVR over the 30‐day monitoring period (Figure 2a–d). BC and UFR were consistently larger in the SCI group compared to the intact group throughout recovery (*p* < 0.001; Figure 2d,f, respectively), while IVI did not differ significantly between groups (Figure 2e). To further characterize recovery dynamics, data were segmented into three post‐injury phases (Herrera et al., 2010; Mimata et al., 1993): an early post‐injury phase (days 3–10), during which involuntary spinally mediated micturition reflexes first emerged; an intermediate phase (days 10–20), characterized by progressive changes in bladder and sphincter function; and a late phase (days 20–30), when lower urinary tract parameters in this model approached a relatively stable pattern (Figure 2a′–f′).

Phase‐specific analysis confirmed a trend of functional improvement in the SCI group across phases. However, even during the chronic phase, VE remained significantly lower (*p* = 0.004), and PVR significantly higher (*p* < 0.001) in the SCI group compared to the intact group (Table 2 and Figure 2b′–c′).

### 3.2 Heterogeneity in chronic phase recovery following SCI

**FIGURE 3:**
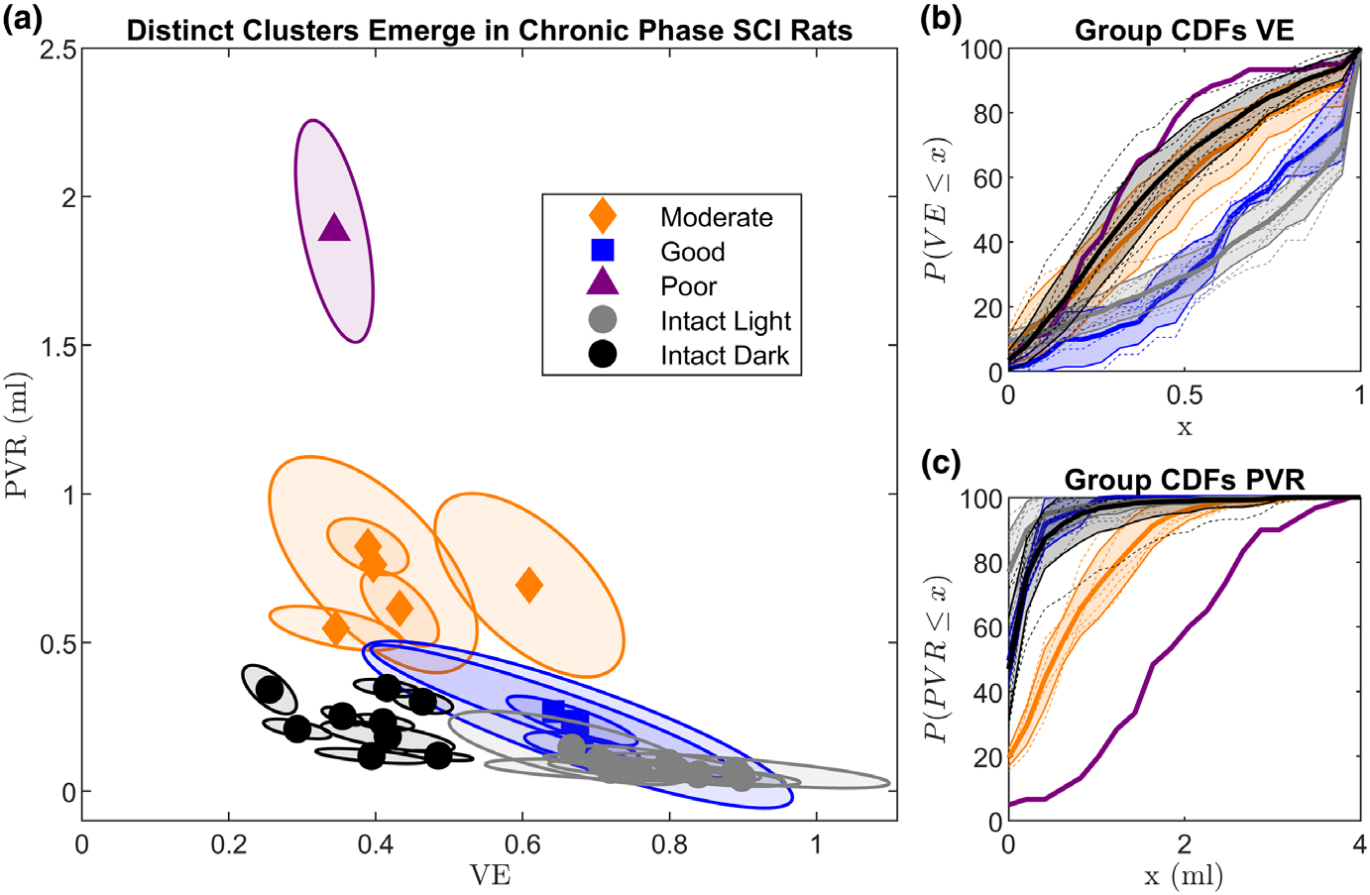
Clustering‐based identification of chronic neurogenic bladder phenotypes. (a) Scatter plot of median post‐void residual (PVR) and median voiding efficiency (VE) during the chronic phase, with animals grouped by unsupervised clustering. Recovery phenotypes are color‐coded: Good (blue), Moderate (orange), and Poor (purple); intact controls are shown for reference during the light cycle (black) and dark cycle (gray). Ellipses represent 90% confidence intervals per animal. (b, c) Cumulative distribution functions (CDFs) for VE (b, top) and PVR (c, bottom) across the intact group and all SCI recovery phenotypes. For each value on the x‐axis, the CDF indicates the proportion of chronic‐phase voids with a VE or PVR less than or equal to that value. Dashed lines represent individual animals; solid lines represent group means; shaded bounds reflect group standard deviation.

Within the SCI group there was substantial variability between rats (see the spread of data within each day–across rats–in Figure 2 in all urodynamic parameters). Given this variability, we wanted to know if any natural recovery phenotypes emerged. We used automated, unsupervised clustering analysis (DBSCAN) to identify potential subgroup phenotypes (Kearney, 2025). Clustering yielded three phenotypes (Figure 3a). For comparison, we include intact rats (black) during the post‐implant period that corresponds to SCI rat chronic phase of recovery, but the intact group was not included in the clustering analysis. Based on the urodynamic parameter values in clustering‐discovered phenotypes, we named them post‐hoc as “good”, “moderate”, and “poor” recovery; blue, 4/10, 0.71 ± 0.03 VE, 0.21 ± 0.06 mL PVR; orange, 5/10, 0.45 ± 0.10 VE, 0.78 ± 0.31 mL PVR; purple, 1/10 0.34 VE, 1.85 mL PVR, respectively. Using cumulative distribution functions (CDFs) to visualize VE and PVR separately further highlights distinct LUT functions between phenotype subgroups (Figure 3b,c). Recovery phenotypes express only levels of VE and PVR. Meaning, rats in the “good recovery” cluster have higher VE and lower PVR, but this does not imply a return to normal LUT function. In fact, the absence of diurnal differences across all SCI phenotypes, in contrast to intact rats (Table 1 and Data S1), indicates disrupted supraspinal input and loss of voluntary control (Gaudet et al., 2018; Herrera & Meredith, 2010), suggesting that voluntary voiding did not return even in the “good recovery” phenotype despite improved voiding efficiency.

**TABLE 1:** Voiding parameters in the Intact and SCI phenotypes across the light and dark cycles.

Variable	Phase	Group	Light cycle (mean ± SD)	Dark cycle (mean ± SD)	Sig
Voided Volume (ml)	Acute	Intact	0.26 ± 0.04	0.17 ± 0.05	**
PVR (ml)	Acute	Intact	0.08 ± 0.035	0.12 ± 0.05	*
VE	Acute	Intact	0.79 ± 0.10	0.63 ± 0.10	*
BC (ml)	Acute	Intact	0.36 ± 0.09	0.29 ± 0.09	ns
IVI (min)	Acute	Intact	45.39 ± 10.55	31.77 ± 5.66	**
UFR (ml/h)	Acute	Intact	0.34 ± 0.08	0.31 ± 0.07	ns
Voided volume (ml)	Intermediate	Intact	0.27 ± 0.04	0.16 ± 0.05	**
PVR (ml)	Intermediate	Intact	0.06 ± 0.023	0.18 ± 0.05	**
VE	Intermediate	Intact	0.82 ± 0.11	0.51 ± 0.07	***
BC (ml)	Intermediate	Intact	0.35 ± 0.05	0.36 ± 0.09	ns
IVI (min)	Intermediate	Intact	39.90 ± 9.26	22.62 ± 3.34	***
UFR (ml/h)	Intermediate	Intact	0.41 ± 0.09	0.48 ± 0.10	ns
Voided volume (ml)	Chronic	Intact	0.08 ± 0.03	0.19 ± 0.06	*
PVR (ml)	Chronic	Intact	0.12 ± 0.03	0.22 ± 0.07	**
VE	Chronic	Intact	0.79 ± 0.15	0.44 ± 0.08	***
BC (ml)	Chronic	Intact	0.40 ± 0.17	0.46 ± 0.14	ns
IVI (min)	Chronic	Intact	35.14 ± 8.84	18.34 ± 4.39	**
UFR (ml/h)	Chronic	Intact	0.45 ± 0.11	0.67 ± 0.13	**
Voided volume (ml)	Acute	SCI	0.21 ± 0.09	0.18 ± 0.10	ns
PVR (ml)	Acute	SCI	1.06 ± 0.55	0.96 ± 0.52	ns
VE	Acute	SCI	0.25 ± 0.21	0.23 ± 0.12	ns
BC (ml)	Acute	SCI	1.40 ± 0.48	1.24 ± 0.60	ns
IVI (min)	Acute	SCI	49.75 ± 13.74	40.32 ± 18.74	ns
UFR (ml/h)	Acute	SCI	0.43 ± 0.10	0.44 ± 0.26	ns
Voided volume (ml)	Intermediate	SCI	0.52 ± 0.12	0.46 ± 0.09	ns
PVR (ml)	Intermediate	SCI	0.80 ± 0.57	0.71 ± 0.47	ns
VE	Intermediate	SCI	0.47 ± 0.16	0.48 ± 0.14	ns
BC (ml)	Intermediate	SCI	1.36 ± 0.63	1.19 ± 0.53	ns
IVI (min)	Intermediate	SCI	55.77 ± 11.36	46.43 ± 8.68	ns
UFR (ml/h)	Intermediate	SCI	0.44 ± 0.14	0.52 ± 0.24	ns
Voided volume (ml)	Chronic	SCI	0.56 ± 0.15	0.55 ± 0.11	ns
PVR (ml)	Chronic	SCI	0.70 ± 0.48	0.71 ± 0.49	ns
VE	Chronic	SCI	0.52 ± 0.16	0.54 ± 0.13	ns
BC (ml)	Chronic	SCI	1.31 ± 0.55	1.30 ± 0.61	ns
IVI (min)	Chronic	SCI	63.12 ± 24.77	59.38 ± 12.27	ns
UFR (ml/h)	Chronic	SCI	0.53 ± 0.10	0.71 ± 0.25	ns

*Note*: Values are presented as mean ± SD. *p* values reflect the comparison between the light and dark cycle within each group, assessed using paired *t*‐tests (*p < 0.05, **p < 0.01, ***p < 0.001).

Abbreviations: BC, bladder capacity; IVI, inter‐void interval; PVR, post‐void residual; UFR, ureter flow rate; VE, voiding efficiency.

Despite the absence of diurnal variation in SCI rats, phenotype‐specific patterns still emerge (Figure 3a–c). VE in the good recovery group approximates values observed during the rest phase (light cycle) in intact animals, typically associated with more complete bladder emptying. In contrast, moderate and poor recovery groups show VE values closer to the active phase (dark cycle), where voiding is less tightly coupled to complete emptying. These patterns suggest differential re‐emergence of spinal, involuntary bladder reflexes across recovery phenotypes rather than restoration of normal, supraspinally regulated LUT function.

### 3.3 Longitudinal analysis of voiding parameters across SCI recovery phenotypes

**FIGURE 4:**
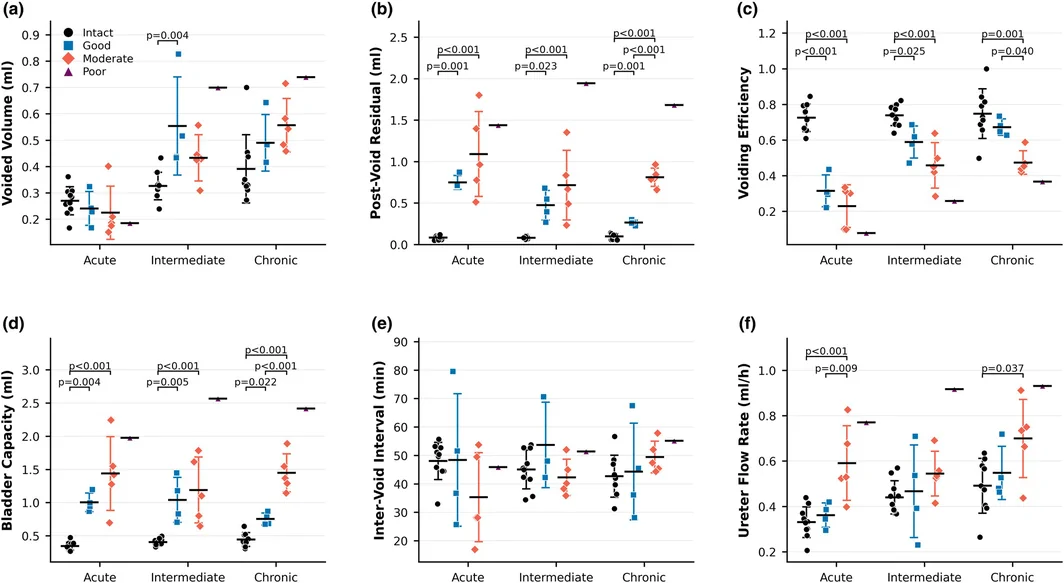
Volume‐derived urodynamic parameters across recovery phases in intact and SCI rats. Voided volume (a), post‐void residual (b), voiding efficiency (c), bladder capacity (d), inter‐void interval (e), and ureter flow rate (f) across the Acute, Intermediate, and Chronic phases. Groups represent intact controls recorded during the light cycle (black circles) and SCI animals classified by recovery phenotype as Good (blue squares), Moderate (orange diamonds), or Poor (purple triangles; *n* = 1). Horizontal lines indicate group means, whiskers indicate ±1 SD, and individual symbols represent per‐animal means. Within each recovery phase, the Intact, Good, and Moderate groups were compared using one‐way ANOVA followed by the Games‐Howell post hoc test; with the Poor group (*n* = 1) excluded from all statistical analyses. Significance was defined as *p* < 0.05.

To evaluate longitudinal differences in LUT recovery, we aggregated urodynamic data by recovery phase across phenotypes (Table 2 and Figure 4). Statistical analysis was not feasible for the poor recovery phenotype (*n* = 1). During the acute phase, all SCI rats showed severely compromised voiding regardless of eventual phenotype (VE <0.2, PVR >0.5 mL; *p* < 0.001 vs. intact for all). By chronic phase, phenotypes diverged significantly. Good recovery rats achieved VE = 0.71 ± 0.03 and PVR = 0.21 ± 0.06 mL, which were not significantly different from intact values (*p* = 0.148 and *p* = 0.285, respectively). Moderate recovery rats showed VE = 0.45 ± 0.10 and PVR = 0.78 ± 0.31 mL (*p* < 0.001 vs. intact for both). The poor recovery rat exhibited VE = 0.34 and PVR = 1.85 mL (Figure 4a,b). IVI showed no significant differences between phenotypes or phases (*p* > 0.05 for all comparisons; Figure 4d).

**TABLE 2:** Comparative analysis of voiding parameters in the Intact group (light cycle) and SCI recovery phenotypes (pooled light and dark cycles) across the three phases (acute, intermediate, and chronic).

Variable	Phase	Group	Mean ± SD	Phenotype	Mean ± SD	Sig	Phenotype	Mean ± SD	Sig	Phenotype	Value
Void volume (ml)	Acute	Intact	0.26 ± 0.04	Good	0.16 ± 0.03		Moderate	0.13 ± 0.10		Poor	0.07
	Intermediate	Intact	0.27 ± 0.04	Good	0.41 ± 0.07	**	Moderate	0.36 ± 0.10		Poor	0.73
	Chronic	Intact	0.28 ± 0.04	Good	0.51 ± 0.15		Moderate	0.54 ± 0.11	*	Poor	0.77
PVR (ml)	Acute	Intact	0.08 ± 0.03	Good	1.13 ± 0.28	**	Moderate	1.17 ± 0.68	**	Poor	1.69
	Intermediate	Intact	0.06 ± 0.02	Good	0.39 ± 0.15	**	Moderate	0.66 ± 0.47	***	Poor	1.99
	Chronic	Intact	0.08 ± 0.03	Good	0.21 ± 0.06	**	Moderate	0.78 ± 0.31	***	Poor	1.85
VE	Acute	Intact	0.79 ± 0.10	Good	0.15 ± 0.04	**	Moderate	0.13 ± 0.07	***	Poor	0.05
	Intermediate	Intact	0.82 ± 0.11	Good	0.55 ± 0.07	**	Moderate	0.41 ± 0.22	***	Poor	0.25
	Chronic	Intact	0.79 ± 0.15	Good	0.71 ± 0.03		Moderate	0.45 ± 0.10	**	Poor	0.34
BC (ml)	Acute	Intact	0.36 ± 0.09	Good	1.27 ± 0.25	**	Moderate	1.47 ± 0.64	***	Poor	1.92
	Intermediate	Intact	0.35 ± 0.05	Good	0.95 ± 0.26	**	Moderate	1.14 ± 0.56	***	Poor	2.56
	Chronic	Intact	0.40 ± 0.17	Good	0.72 ± 0.12	**	Moderate	1.52 ± 0.39	***	Poor	2.61
IVI (min)	Acute	Intact	45.39 ± 10.55	Good	31.20 ± 22.66		Moderate	18.39 ± 8.31		Poor	37.76
	Intermediate	Intact	39.90 ± 9.26	Good	37.53 ± 12.93		Moderate	39.90 ± 13.00		Poor	47.97
	Chronic	Intact	35.14 ± 8.84	Good	44.76 ± 25.17		Moderate	45.46 ± 10.67		Poor	48.29
UFR (ml/h)	Acute	Intact	0.34 ± 0.08	Good	0.39 ± 0.08		Moderate	0.55 ± 0.24	**	Poor	0.54
	Intermediate	Intact	0.41 ± 0.09	Good	0.37 ± 0.13		Moderate	0.53 ± 0.12		Poor	0.84
	Chronic	Intact	0.45 ± 0.11	Good	0.51 ± 0.06		Moderate	0.69 ± 0.26		Poor	0.96

*Note*: Values are presented as mean ± SD. Statistical comparisons among the Intact, Good, and Moderate groups were performed using one‐way ANOVA with Tukey's post‐hoc test for multiple comparisons (*p < 0.05, **p < 0.01, ***p < 0.001). The Poor phenotype contained only one animal and was therefore excluded from statistical analysis.

Abbreviations: BC, bladder capacity; IVI, inter‐void interval; PVR, post‐void residual; UFR, ureter flow rate; VE, voiding efficiency.

Good recovery BC (0.22 ± 0.04 mL chronic) approximated intact values (0.18 ± 0.03 mL, *p* = 0.198), while moderate (0.89 ± 0.15 mL) and poor (2.06 mL) phenotypes showed persistently elevated BC (*p* < 0.001) across all phases (Figure 4c). Good recovery UFR (0.012 ± 0.003 mL/min) matched intact values (0.010 ± 0.002 mL/min, *p* = 0.412), while moderate (0.018 ± 0.005 mL/min, *p* = 0.031) and poor (0.021 mL/min) showed sustained elevation (Figure 4e).

Taken together, good recovery showed continuous improvement throughout; moderate recovery improved from the acute to intermediate phase but plateaued thereafter, whereas poor recovery showed progressive decline.

### 3.4 Longitudinal analysis of pressure parameters across SCI recovery phenotypes

**FIGURE 5:**
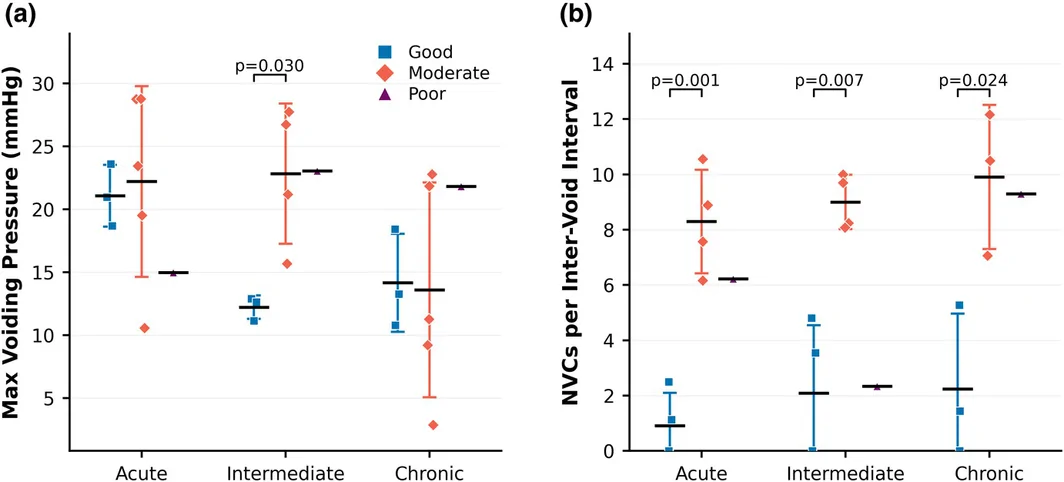
Pressure‐derived urodynamic parameters across recovery phases in SCI rats. Maximum voiding pressure (mmHg; a) and the average number of non‐voiding contractions (NVCs) per inter‐void interval (b) across the Acute, Intermediate, and Chronic phases. Groups represent SCI animals classified by recovery phenotype: Good (blue square), Moderate (orange diamond), and Poor (purple triangle; *n* = 1). Colored whiskers represent the group mean ± SD (horizontal black tick = mean); individual points represent per‐animal means. Within each phase, the Good and Moderate groups were compared using Welch's ANOVA followed by the Games‐Howell post hoc test, with the Poor group (*n* = 1) excluded from all statistical analyses. Significance was defined as *p* < 0.05.

Maximum voiding pressure and non‐voiding contraction frequency were compared between recovery phenotypes at each phase (Figure 5). Maximum voiding pressure did not differ between phenotypes during the acute phase. By the intermediate phase, the good recovery phenotype had the lowest pressures (12.2 ± 0.8 mmHg) and differed significantly from moderate recovery (22.8 ± 5.5 mmHg, *p* = 0.030). During the chronic phase, phenotype differences were no longer significant, though the poor recovery rat maintained the highest pressures (Figure 5a).

NVC counts separated the phenotypes at every phase. Good recovery animals exhibited significantly fewer NVCs per inter‐void interval than moderate recovery animals during the acute (0.9 ± 1.2 vs. 8.3 ± 1.9, *p* = 0.001), intermediate (2.1 ± 2.5 vs. 9.0 ± 0.9, *p* = 0.007), and chronic phases (2.2 ± 3.1 vs. 9.9 ± 2.6, *p* = 0.024) (Figure 5b). The single poor recovery animal did not follow a monotonic course, falling between the two groups at the acute phase, within the good recovery range at the intermediate phase, and within the moderate recovery range by the chronic phase. Inter‐void interval did not differ significantly between good and moderate recovery phenotypes at any phase (acute: 48.4 ± 23.3 vs. 35.3 ± 15.7 min, *p* = 0.35; intermediate: 53.7 ± 15.0 vs. 42.3 ± 6.4 min, *p* = 0.18; chronic: 44.3 ± 17.0 vs. 49.4 ± 5.6 min, *p* = 0.72; Figure 3e and Table 2). Differences in NVC counts per interval therefore primarily reflect contraction frequency rather than filling time.

### 3.5 Predicting chronic phase VE from acute phase voiding parameters

Forecasting long‐term LUT function after SCI from data available early in the recovery process could improve precision treatment planning; therefore, we asked if a rat's chronic‐phase VE is predictable from acute‐phase urodynamic parameters. Using GLM with a logit link function (to constrain predictions to the same 0–1 interval as VE), we identified the best performing set of predictors (see methods) as follows:

$${\tilde{\mathrm{VE}}}_{c}\approx \frac{e^{p_{0}+p_{1}{\tilde{\mathrm{VV}}}_{a}{\tilde{\mathrm{VE}}}_{a}+p_{2}{\tilde{\mathrm{UFR}}}_{a}{\tilde{\mathrm{VE}}}_{a}^{2}}}{1+e^{p_{0}+p_{1}{\tilde{\mathrm{VV}}}_{a}{\tilde{\mathrm{VE}}}_{a}+p_{2}{\tilde{\mathrm{UFR}}}_{a}{\tilde{\mathrm{VE}}}_{a}^{2}}}$$

where ${\tilde{\mathrm{VE}}}_{c}$ is the median chronic‐phase void efficiency, ${\tilde{\mathrm{VV}}}_{a}$ is the median acute‐phase voided volume, ${\tilde{\mathrm{VE}}}_{a}$ is the median acute‐phase void efficiency, ${\tilde{\mathrm{UFR}}}_{a}$ is the median acute‐phase natural bladder filling rate and p are fit model parameters (values in Table 3). On the fit data, this model explained >90% of the variance (*R* ^2^ = 0.92) and was statistically significant compared to the constant model ($F_{2,7}=36.1$, *p* < 0.001).

**TABLE 3:** Parameters of the Generalized Linear Model. The model coefficients were all statistically significant (*p* < 0.01) and the model captured most of the variance ($R^{2}=0.92$) in the rats' median chronic Void Efficiency (${\tilde{\mathrm{VE}}}_{c}$).

Parameter	Estimate	Standard error	*t*‐statistic	*p*‐value
*p*_0_	−0.71	0.12	−6.00	<0.001
*p*_1_	75.81	13.10	5.79	<0.001
*p*_2_	−3.21 * 10^5^	7.47 * 10^4^	−4.29	*p* = 0.004

**FIGURE 6:**
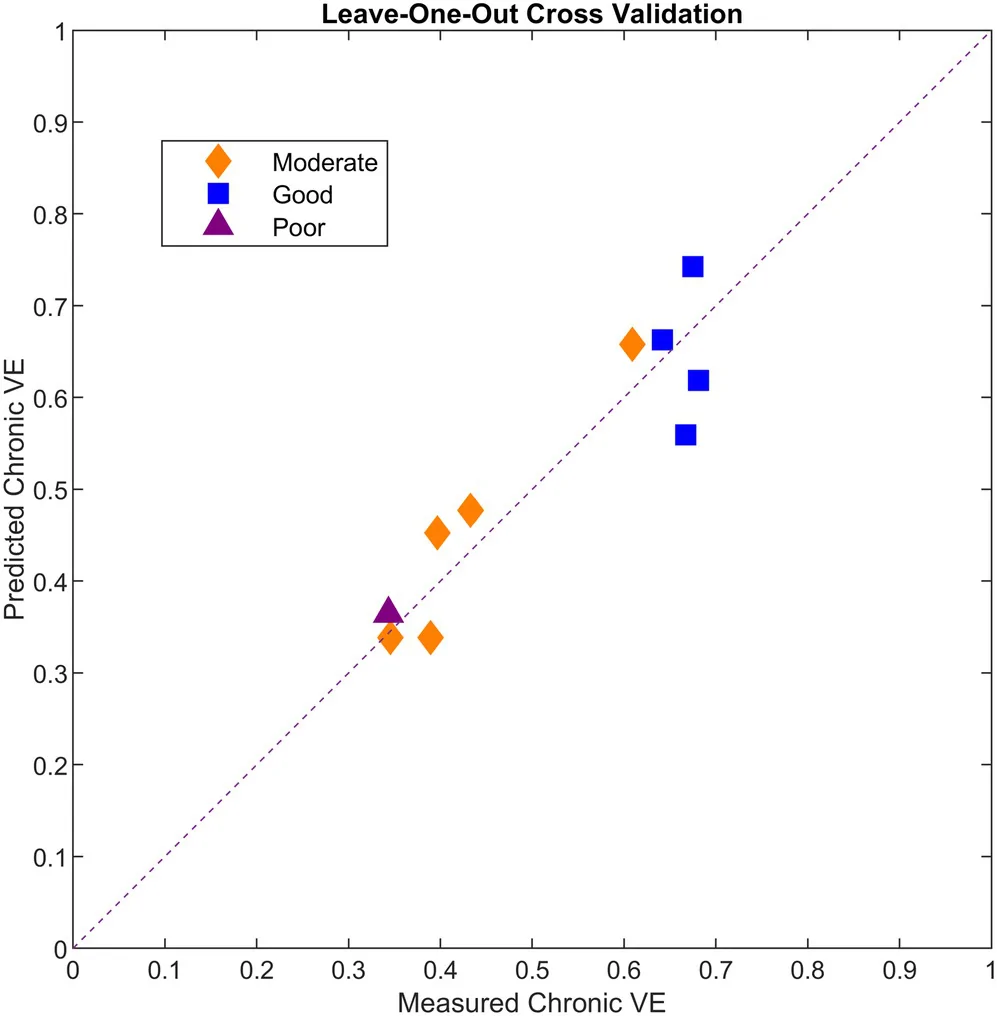
Leave‐one‐out cross‐validation of chronic voiding efficiency predictions. Each point represents one withheld animal, with model‐predicted median chronic VE plotted against measured chronic VE. Points are color‐coded by recovery phenotype: Good (blue), Moderate (orange), and Poor (purple); phenotype classification was not used in model prediction. The dashed line indicates the line of identity (predicted = measured). Predicted and measured voiding efficiency were significantly correlated (*R* ^2^ = 0.84, *p* < 0.001).

To assess the model's generalizability, we re‐fit the model an additional 10 times, each time leaving out one rat, then compared the model's prediction for the withheld rat to its actual 𝑉𝐸. This approach let us validate the model despite our relatively small sample size. The 10 versions of the model accurately predicted the withheld 10th rat's 𝑉𝐸 (Figure 6, leave one out cross‐validation, LOOCV), suggesting that the model is not overfitting. Rats lying near the 45° line (Figure 6) indicate that model predictions on left‐out examples closely matched actual 𝑉𝐸 across all three recovery phenotypes (*R* ^2^ = 0.84, *p* < 0.001). The parameter values had low variation across this cross‐validation procedure, suggesting that this model is relatively stable and not idiosyncratic to this specific dataset ($-0.71\pm 0.03,75.78\pm 3.9,-3.20\left(10^{5}\right)\pm 2.32\left(10^{4}\right)$, for $p_{0},p_{1},$ and $p_{2}$).

### 3.6 Visualizing the trajectory of urodynamics across recovery using principal components analysis (PCA)

**FIGURE 7:**
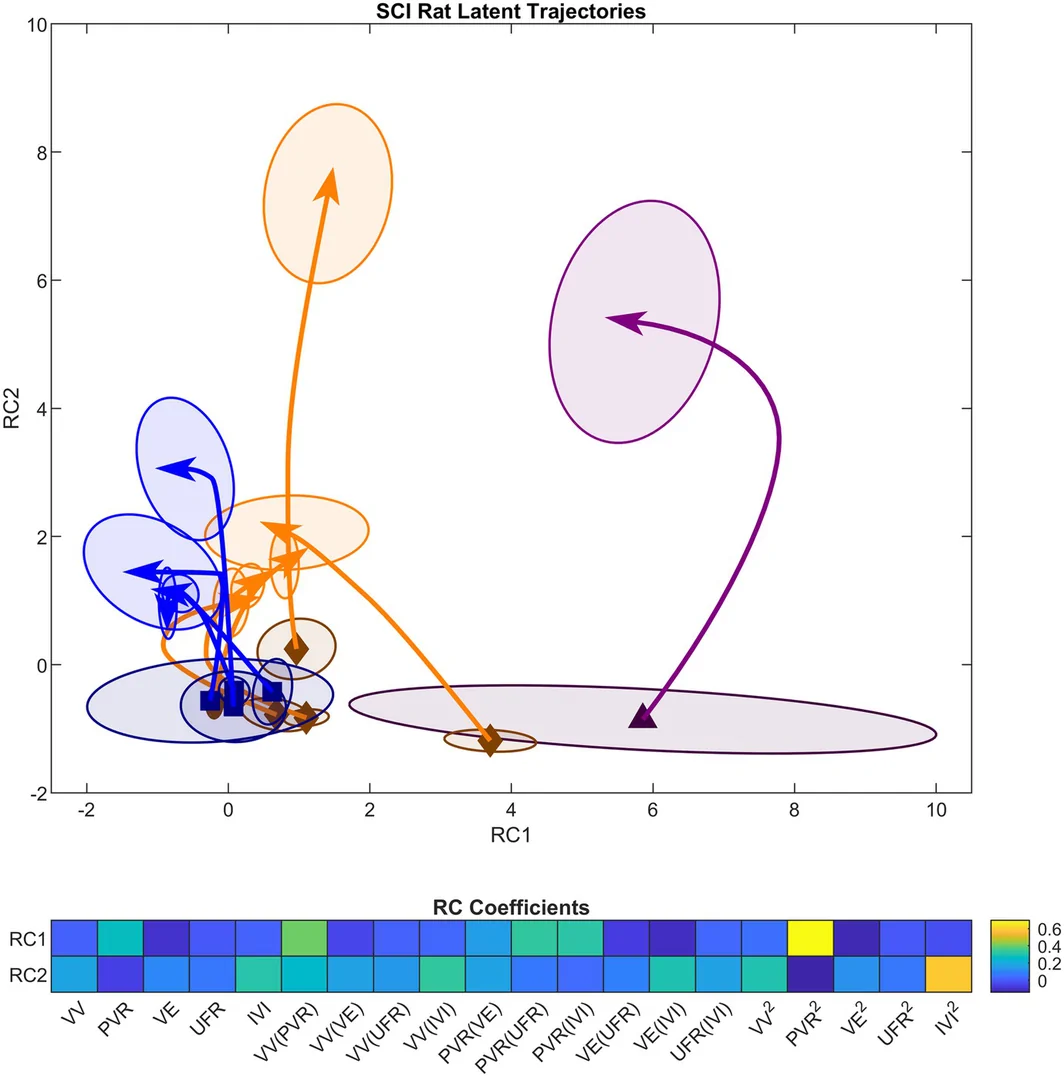
Latent urodynamic trajectories across recovery phenotypes derived from principal component analysis. Each trajectory represents the change in urodynamic function over time for an individual animal, from the acute to chronic phase (arrows). Animals are distributed along varimax‐rotated principal components RC1 and RC2, with the Good (blue), Moderate (orange), and Poor (purple) phenotypes clustering separately. RC1 primarily captures post‐void residual, voiding efficiency, ureter flow rate, and inter‐void interval; RC2 primarily captures voided volume and inter‐void interval. Ellipses represent 90% confidence intervals for the median RC scores per phenotype.

Plotting urodynamic parameters individually throughout recovery helps us understand specific aspects of LUT function but understanding how the entire LUT evolves is a challenge because there are so many parameters changing simultaneously (e.g. Figure 3). PCA offers a tractable way to visualize all parameter changes at once by identifying the most salient changes of linear combinations of all urodynamic parameters simultaneously. Those linear combinations (the so‐called principal components, PCs) can be rotated to increase their interpretability (by reducing the overlap of urodynamic parameters contributing to each PC) without changing their combined representation. In Figure 7a we plot the changes throughout recovery of each rat along the varimax rotated principal components (RCs), which enabled us to examine (1) what were the major sources of variation among all the median urodynamic parameters, and (2) to what extent each urodynamic parameter contributes to those sources.

The median urodynamic parameters of each SCI rat begin at the dot and evolve through time along the path indicated by the arrow (Figure 7b). Interestingly, rats with different chronic phase phenotypes (colors) appear somewhat separate in the PC space, with much of the separation occurring along the RC1 dimension (representing lower efficiency and higher void residual volume). Changes through time appear mainly confined to the RC2 dimension (representing increased VE, IVI, VV, and decreased PVR). Thus, the unsupervised PCA analysis suggests that the two most dominant modes of urodynamic parameter variability are associated with phenotypes and processes related to recovery. It also suggests that an increased inter‐void interval is a recovery‐associated parameter for all phenotypes.

## 4 DISCUSSION

Continuous 24/7 urodynamic monitoring across more than 5000 voids over 4 weeks in a complete T9 transection rat model showed that neurogenic bladder function follows a time‐dependent but highly heterogeneous trajectory after SCI. During the acute phase, adverse urodynamic features, including detrusor overactivity, elevated maximal detrusor pressure during filling, reduced voiding efficiency, and increased urinary retention, were consistently observed across animals. Despite this early phenotypic similarity, long‐term outcomes diverged substantially, ranging from low‐risk neurogenic bladder development with efficient voiding to high‐risk phenotypes with poor voiding. A GLM based on acute‐phase multivariate urodynamic parameters accurately predicted chronic voiding phenotype (*R* ^2^ = 0.90, *p* < 0.001), supporting the prognostic value of early LUT dynamics and suggesting a window for targeted intervention before irreversible dysfunction develops.

### 4.1 Heterogeneous recovery trajectories emerge from seemingly identical acute dysfunction

While pooled analysis of all SCI rats showed gradual functional improvement post‐SCI (Figure 2b,c), unsupervised clustering revealed three distinct recovery phenotypes (Figure 3). This heterogeneity parallels clinical observations, where individuals with comparable injury classifications exhibit variable LUT recovery trajectories, making early prognosis challenging (Saito et al., 2021; Wyndaele, 2016).

All SCI rats showed complete voiding dysfunction during the acute phase regardless of eventual chronic phenotype (Table 2). VE and PVR were statistically indistinguishable across groups, consistent with established pathophysiology after spinal shock, in which reorganization of spinal reflex circuits following loss of supraspinal control remains insufficient to restore normal voiding (Ahmed et al., 2006; de Groat & Yoshimura, 2006). By the intermediate phase, divergent patterns emerged: good recovery rats showed continuous improvement in VE and reduction in PVR; moderate recovery rats demonstrated partial improvement that plateaued; and the poor recovery rat exhibited progressive functional deterioration. This uniform acute impairment followed by phenotype divergence represents the core prognostic challenge; all animals appear similarly impaired initially yet follow substantially different recovery trajectories.

### 4.2 Multivariate early urodynamic assessment predicts long‐term bladder recovery

By applying a GLM framework to acute phase urodynamic parameters, we identified early indicators predictive of long‐term recovery phenotypes (*R* ^2^ = 0.90, *F* = 33.7, *p* < 0.001) (Figure 6). This predictive power emerged from parameter interactions rather than individual measurements, which may help explain why conventional clinical assessment evaluating parameters independently has limited early prognostic value.

The interaction of acute VV and VE (VVa·VEa) was a significant positive predictor of chronic VE (*β* = 75.81, *p* < 0.001), indicating that the relationship between voided volume and efficiency early after injury carries prognostic information about long‐term voiding capacity. The negative association of UFRa·VEa^2^ (*β* = −3.21 × 10^5^, *p* = 0.004) further highlights the prognostic relevance of storage parameters: elevated ureter flow rate during the acute phase, when coupled with voiding inefficiency, was associated with poorer chronic outcomes. These findings are broadly consistent with clinical studies identifying early biomarkers associated with differential LUT recovery (Ferbert et al., 2017; Gazzolo et al., 2004; Heppner et al., 2022; Redmond et al., 2019), though direct mechanistic parallels remain to be established.

There is an apparent tension between our longitudinal observations and our predictive model: if acute phase urodynamic parameters were not statistically different between phenotype groups (Table 2), how did the GLM use acute data to accurately predict chronic outcomes? The projection of urodynamic parameters into PC space (Figure 7) offers a resolution. The acute phase represents one of the dominant variance modes across urodynamic parameters (RC1), and the starting position of each rat along this dimension correlates with chronic phenotype, despite the lack of significant group differences on individual parameters. RC2 mainly captures within‐animal changes across recovery phases, improved efficiency, longer IVIs, lower PVRs, reflected in the predominantly vertical paths in Figure 7. Recovery trajectories are therefore not determined by individual parameters in isolation, but by multivariate combinations that reveal underlying dysfunction patterns. The good predictive accuracy across multiple parameter subsets also implies considerable redundancy in the parameter space, suggesting that a carefully chosen subset would be sufficient for prediction, which simplifies requirements for any future diagnostic application.

### 4.3 Storage parameters are phenotype‐specific from the acute phase and remain stable across recovery

Storage and voiding parameters revealed distinct temporal dynamics, reflecting the early urodynamic interactions identified by GLM as predictors of chronic outcome. Voiding parameters (VE, PVR) showed progressive, time‐dependent changes that diverged across phenotypes throughout all phases. Storage parameters (bladder capacity, UFR), by contrast, established phenotype‐specific values early and remained stable thereafter. Bladder capacity differed significantly across phenotypes, smallest in good recovery, largest in poor recovery, and was consistent within each phenotype across phases. UFR mirrored this pattern: good recovery rats maintained low UFR approximating intact levels throughout, while moderate and poor phenotypes showed persistently elevated UFR (Figure 4 and Figure S2).

This early stabilization has an important implication: if storage parameters are already phenotype‐specific in the acute phase, before involuntary reflex voiding reorganized. They are unlikely to result from voiding pathway reorganization and instead likely reflect bladder wall properties established at or shortly after injury. The bladder undergoes rapid and substantial change in the acute phase: uroepithelial barrier failure, tight junction protein loss, and neutrophil infiltration occur within hours of SCI (Herrera et al., 2010), while tissue remodeling genes including tropoelastin, lysyl oxidase, and TGF‐β1 are massively upregulated within days, alongside activation of inflammatory and immune pathways (Wognum et al., 2009). These rapid structural and molecular changes in the bladder wall could plausibly shape compliance, mechanosensitivity, and afferent signaling thresholds in a phenotype‐specific manner and may influence how voiding function recovers over time (de Groat & Yoshimura, 2009; de Groat & Yoshimura, 2010; Rong et al., 2002). UFR, estimated here from bladder capacity divided by IVI, reflects urine production rate determined by renal filtration rather than bladder physiology properly; its phenotype specifically from the acute phase suggest that systemic physiological differences at the time of injury may contribute alongside local bladder changes.

Taken together, these observations point to two processes operating on different timescales. Storage characteristics are set early and remain fixed, consistent with bladder wall properties established at or shortly after injury (Herrera et al., 2010); voiding function evolves over weeks, consistent with progressive reorganization of spinal reflex circuitry (de Groat & Yoshimura, 2009; de Groat & Yoshimura, 2010). The good‐recovery phenotype accordingly reflects more robust reorganization of spinal voiding circuits rather than restored supraspinal regulation. This distinction matters for interpretation: although animals in the good recovery phenotype recover efficient emptying, that efficiency is produced by an involuntary spinal reflex resembling an immature voiding pattern, so comparable voiding efficiency can mask a fundamental difference in the mechanism of micturition. Because the acute‐phase parameter interactions identified by the GLM draw on both storage and voiding measures, they plausibly index both the capacity for spinal reorganization and the emerging mechanical properties of the bladder wall. Their predictive value for long‐term phenotype may therefore arise from either process or both. Separating the two would require measures that dissociate them; bladder wall compliance or afferent signaling assessed independently of voiding and remains an important goal for future work.

### 4.4 Loss of diurnal voiding variation as a marker of recovery phenotype after SCI

Intact rats exhibited clear diurnal variation in voiding behavior, with higher VE and lower PVR during the light (rest) cycle relative to the dark (active) cycle (Table 1; Figure 3; Figure S1). Following SCI, this variation was abolished and did not recover across the 4‐week observation period, a finding consistent with disrupted supraspinal control of voiding (Gaudet et al., 2018; Herrera & Meredith, 2010). The comparisons across 3 post‐SCI phenotypes showed a phenotype‐specific stabilization pattern. SCI rats in good phenotype achieved VE values comparable to intact rats during the light cycle (0.71 ± 0.03), while moderate and poor phenotypes stabilized at levels resembling the intact dark cycle (0.45 ± 0.10 and 0.34, respectively). The persistent absence of diurnal variation, alongside phenotype specific VE stabilization, aligns with our earlier finding that storage parameters, including UFR, differ by recovery phenotype in the acute phase and stabilize afterward. This is consistent with previous findings, showing that the diurnal pattern is partly regulated by vasopressin, which normally exhibits a circadian rhythm that suppresses urine production during sleep, reducing nocturnal voiding frequency and coordinating upper and lower urinary tract functions (Boone & Deen, 2008; Gizowski et al., 2017; Verbalis, 2014). Loss of supraspinal vasopressin regulation following SCI has been associated with disrupted diurnal urine production and polyuria in both humans and rodent models (Gumbel et al., 2021; Osei‐Owusu et al., 2022), which in turn imposes an increased filling load on the LUT independent of detrusor or sphincter dysfunction. Notably, our findings showed that although all SCI animals exhibited elevated UFR relative to intact rats, urine production still varied systematically across phenotypes, suggesting that differential upper urinary tract output may be partly regulated by local or segmental pathways and could serve as an early biomarker of bladder reorganization capacity, though the precise mechanisms remain to be determined.

## 5 LIMITATIONS AND FUTURE DIRECTIONS

These findings are preliminary and hypothesis‐generating. Validation in larger cohorts and with clinically accessible urodynamic measures is required to establish generalizability and prognostic utility. Only female rats were studied to reduce biological variability; however, known sex differences in SCI‐related bladder dysfunction and distinct male urodynamic profiles may influence phenotypic distribution and predictive interactions (Myers et al., 2023; Stewart et al., 2020). Validation in male and mixed‐sex cohorts is therefore necessary. Continuous housing in metabolic cages precludes concurrent external urethral sphincter (EUS) electromyography (EMG), limiting electrophysiological confirmation of detrusor–sphincter dyssynergia. Future studies incorporating simultaneous EUS EMG will enable more precise assessment of sphincter coordination.

## 6 CONCLUSION

This study provides, to our knowledge, the first continuous longitudinal characterization of post‐SCI bladder reorganization in a rat model. No single acute urodynamic parameter reliably predicted chronic neurogenic bladder phenotype; instead, a GLM integrating multivariate interactions among early urodynamic features achieved high predictive accuracy for long‐term voiding outcomes. These findings show that recovery of bladder control after SCI is highly variable and cannot be explained by injury level alone but rather reflects the pattern of spinally mediated bladder reorganization. Importantly, this trajectory appears to be established early during the acute recovery phase, likely through the combined effects of spinal neuroplasticity and peripheral remodeling, including bladder wall adaptation, smooth muscle changes, and altered afferent signaling. Together, these results support the use of predictive models based on acute‐phase urodynamic profiles to identify individuals at risk for adverse chronic outcomes and to guide early, personalized intervention before dysfunction becomes established.

## AUTHOR CONTRIBUTIONS

**Behnaz Afrashteh:** Formal analysis; software; visualization. **Steafan Khan:** Formal analysis; visualization. **Rabeya Zinnat Adury:** Data curation; methodology; validation. **Damiano Angoli:** Data curation. **Zachary C. Danziger:** Conceptualization; data curation; formal analysis; funding acquisition; investigation; methodology; project administration; resources; software; supervision.

## FUNDING INFORMATION

This study was supported by the Craig H Neilsen Foundation (pilot award pC ID 460399) and NIH R01DK133605.

## CONFLICT OF INTEREST STATEMENT

The author(s) have no competing interests to disclose.

## Supporting information


**Figure S1:** Light/dark cycle comparison of voiding urodynamic parameters in intact and SCI rats. (a–f) Intact group; (a′–f′) SCI group. Time course of urodynamic parameters over 30 days post‐SCI (and 25 comparable days in intact animals), comparing the light cycle (orange) and dark cycle (black). Data are expressed as mean ± SD.
**Figure S2:** Percent deviation in ureter flow rate relative to intact controls across recovery phases. ΔUFR (%) represents the per‐animal deviation from the intact group mean, calculated as [(SCI value − intact mean)/intact mean] × 100. The shaded gray band indicates the ±1 SD range of intact UFR values and serves as the normal reference range; the dashed line at 0% indicates the intact reference level. Groups represent SCI animals classified by recovery phenotype: Good (blue square), Moderate (orange diamond), and Poor (purple triangle; *n* = 1). Colored whiskers represent the group mean ± SD (horizontal black tick = mean); individual points represent per‐animal means. Within each phase, the Good and Moderate groups were compared using Welch's ANOVA followed by the Games‐Howell post hoc test; with the Poor group (*n* = 1) excluded from all statistical analyses. Significance was defined as *p* < 0.05.

## Data Availability

The datasets analyzed during the current study are available from the corresponding author upon reasonable request.
