# Supplementary material for: Early urodynamic profiles predict lower urinary tract recovery after spinal cord injury in female rats
Source: Physiol Rep. 2026 Sep 9;14(17):e71101. doi: 10.14814/phy2.71101 (PMC13559020; doi:10.14814/phy2.71101)
Supplement: Supplementary file 1 [file PHY2-14-e71101-s001.docx]

## Supplementary Figures

**Supplementary Figure 1.** Light/dark cycle comparison of voiding urodynamic parameters in intact and SCI rats. (A-F) Intact group; (A′-F′) SCI group. Time course of urodynamic parameters over 30 days post-SCI (and 25 comparable days in intact animals), comparing the light cycle (orange) and dark cycle (black). Data are expressed as mean ± SD.

 **Supplementary Figure 2.** Percent deviation in ureter flow rate relative to intact controls across recovery phases. ΔUFR (%) represents the per-animal deviation from the intact group mean, calculated as [(SCI value − intact mean) / intact mean] × 100. The shaded gray band indicates the ± 1 SD range of intact UFR values and serves as the normal reference range; the dashed line at 0% indicates the intact reference level. Groups represent SCI animals classified by recovery phenotype: Good (blue square), Moderate (orange diamond), and Poor (purple triangle; n = 1). Colored whiskers represent the group mean ± SD (horizontal black tick = mean); individual points represent per-animal means. Within each phase, the Good and Moderate groups were compared using Welch's ANOVA followed by the Games-Howell post hoc test; with the Poor group (n = 1) excluded from all statistical analyses. Significance was defined as p < 0.05.
